# Regenerative potential of multinucleated cells: bone marrow adiponectin-positive multinucleated cells take the lead

**DOI:** 10.1186/s13287-023-03400-w

**Published:** 2023-07-04

**Authors:** Shiva Moein, Naser Ahmadbeigi, Rezvan Adibi, Sara Kamali, Kobra Moradzadeh, Pardis Nematollahi, Nance Beyer Nardi, Yousof Gheisari

**Affiliations:** 1 Regenerative Medicine Research Center, Isfahan University of Medical Sciences, Isfahan, 8174673461 Iran; 2 Department of Genetics and Molecular Biology, Isfahan University of Medical Sciences, Isfahan, Iran; 3 Gene Therapy Research Center, Digestive Diseases Research Institute, Tehran University of Medical Sciences, Tehran, Iran; 4 Department of Pathology, Faculty of Medicine, Isfahan University of Medical Sciences, Isfahan, Iran; 5 Institute of Cardiology of Rio Grande do Sul, Av Princesa Isabel 370, Porto Alegre, RS 90620-001 Brazil

**Keywords:** Bone marrow regeneration, Multinucleation, Irradiation, Adipocyte, Adiponectin, Hematopoiesis

## Abstract

**Background:**

Polyploid cells can be found in a wide evolutionary spectrum of organisms. These cells are assumed to be involved in tissue regeneration and resistance to stressors. Although the appearance of large multinucleated cells (LMCs) in long-term culture of bone marrow (BM) mesenchymal cells has been reported, the presence and characteristics of such cells in native BM and their putative role in BM reconstitution following injury have not been fully investigated.

**Methods:**

BM-derived LMCs were explored by time-lapse microscopy from the first hours post-isolation to assess their colony formation and plasticity. In addition, sub-lethally irradiated mice were killed every other day for four weeks to investigate the histopathological processes during BM regeneration. Moreover, LMCs from GFP transgenic mice were transplanted to BM-ablated recipients to evaluate their contribution to tissue reconstruction.

**Results:**

BM-isolated LMCs produced mononucleated cells with characteristics of mesenchymal stromal cells. Time-series inspections of BM sections following irradiation revealed that LMCs are highly resistant to injury and originate mononucleated cells which reconstitute the tissue. The regeneration process was synchronized with a transient augmentation of adipocytes suggesting their contribution to tissue repair. Additionally, LMCs were found to be adiponectin positive linking the observations on multinucleation and adipogenesis to BM regeneration. Notably, transplantation of LMCs to myeloablated recipients could reconstitute both the hematopoietic system and BM stroma.

**Conclusions:**

A population of resistant multinucleated cells reside in the BM that serves as the common origin of stromal and hematopoietic lineages with a key role in tissue regeneration. Furthermore, this study underscores the contribution of adipocytes in BM reconstruction.

**Graphical Abstract:**

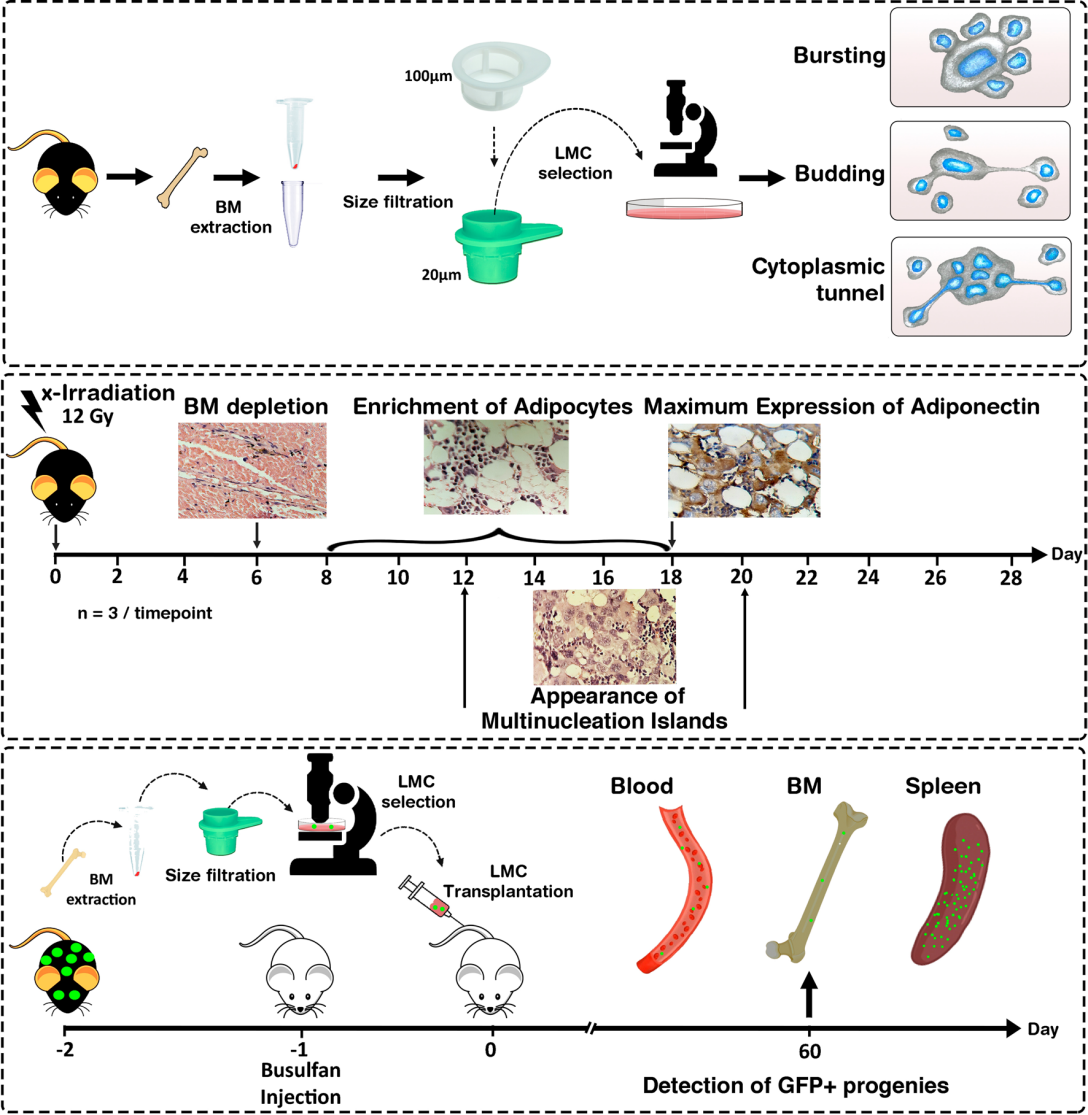

**Supplementary Information:**

The online version contains supplementary material available at 10.1186/s13287-023-03400-w.

## Introduction

Recent achievements in the field of regenerative medicine and cell therapy denote a paradigm shift toward the treatment of complex disorders [1]. A perspective issue in this field is activating tissue-resident stem cells to cure disease complications [2]. Nevertheless, the current knowledge on the origin and function of these cells in mammalian organs is still insufficient.

Our recent findings indicate that bone marrow (BM)-derived mesenchymal stromal cells (MSCs) acquire a multinucleation phenotype and produce mononucleated progenies via budding or bursting [3]. Furthermore, our preliminary studies determined the formation of tubule-like structures and expression of CD31 by these large multinucleated cells (LMCs) [4]. On the other hand, size filtration demonstrated the presence of structural aggregates in the BM which not only produce a substantial number of colony-forming unit fibroblasts in the culture but also have a high regenerative capability to reconstitute BM after sub-lethal irradiation [5]. These findings supported the hypothesis on the beneficial effects of cell polyploidization on regenerative potential of tissues to produce various cell types in a short period of time.

Although polyploidy is generally believed to be more linked to abnormality, the relation of genome content and adaptation is demonstrated in a wide spectrum of species. Yeasts comprise a polyploidy state in response to different types of stressors [6–8] to augment gene expression capacity and tolerate the loss of function mutations [9]. On the other hand, polyploidy is a common phenotype among plants resulting in not only more product yield but also adaptive responses to environmental changes [10]. Drosophila augments the genome content in various tissues as a reparative mechanism to compensate for cell loss [11–14]. In addition, recent findings of Cao et al. determined that the epicardium of the zebrafish forms zones of multinucleation to activate regenerative responses to mechanical tensions [15]. Mammalians also increase ploidy levels during regenerative processes in various organs. The liver encompasses more than 50% polyploid cells [16, 17], which increase upon hepatectomy and different acute and chronic liver injuries [18–20]. These polyploid hepatocytes can enter reductive division and produce a lot of progenies to regenerate the injured tissue [21]. Based on Lazzeri et al.’s findings, Pax8-positive epithelial cells in the distal tubules of the kidney acquire a polyploid phenotype in response to ischemic injury [22]. Keratinocytes [23], retinal pigment epithelial cells [24], mammary gland epithelial cells [25], osteoclasts [26], trophoblast giant cells [27], and megakaryocytes [28] are the other cell types whose polyploidization has been reported during tissue hemostasis and repair.

Polyploidy is also a remarkable phenotype in diverse cancer types. Polyploid giant cancer cells (PGCCs) are putative sources of cancer stem cells and modulators of tumor recurrence post-therapy [29]. A wide variety of cell types such as CD31^+^ endothelial cells [30], erythrocytes [31], macrophages, neutrophils [30, 32], and tumor-associated fibroblasts [33] are originated from these polyploid cells, determining their high plasticity. Furthermore, these cells are the source of tumor heterogeneity and tolerance against environmental stressors as well as cancer therapeutics. In a recent review, we proposed an analogy between the function of polyploid cells in cancer and regeneration [29]. In line with this concept, we found zoledronic acid that is an osteoclast targeting agent to be effective in eradicating PGCCs originated from bladder cancer cells [34].

Based on the above-mentioned studies and our previous findings, it can be hypothesized that BM-derived LMCs have a high plasticity and a significant contribution to tissue regeneration. To cover this issue, the morphological features of LMCs were assessed post-BM extraction. Moreover, an optimized approach was developed for the single isolation of these cells. Afterward, the differentiation potential of isolated LMCs into different lineages was investigated by transplantation into myeloablated mice. This study underscores the critical role of multinucleated cells in the regeneration process.

## Materials and methods

### Mice BM ablation

Normal 6–8-week-old C57BL/6 mice were obtained from the Royan Institute (Isfahan, Iran). For myeloablation, mice were anesthetized by ketamine and xylazine (Alfasan, Netherland) at the dose of 115 and 11.5 mg/kg, respectively. They were then treated with an X-irradiation dose 12 Gy using ONCOR instrument (Siemens, Germany) at Isfahan Milad Hospital. To reduce the mortality rate, irradiation was performed three times at 4-h intervals. A maximum of five mice were kept per each standard cage. Infection was avoided by adding oral enrofloxacin (0.5 mL/L, Advacare, India) to the water. Also, injectable enrofloxacin (Bayer Hispania, Spain) was administrated subcutaneously at the dose of 5 mg/kg every 12 h for 3 days. Two, 4, 6, 8, 10, 12, 14, 16, 18, 20, 22, 24, 26, and 28 days after irradiation, three mice were killed. A total number of 60 mice were considered for irradiation, and the mice that showed signs of lethargy during the study were excluded. Also, in each time interval mice were randomly selected from three different cages. The right femur and the left femur were fixed in buffered formalin 10% (Sigma, Germany). One tibia of the irradiated mice was used for BM isolation and cell culture. The sections were captured by an optical KF2 Carl Zeiss microscope (Carl Zeiss, Germany) equipped with × 10, × 40, and × 100 magnification objective lens. Histopathology scoring for cellularity, regeneration, multinucleation, and adipose tissue was performed in a double-blind manner according to Additional file 1: Table S1. Briefly, according to our observations, 30–70% of the marrow is occupied by hematopoietic cells in normal mice. For each 10% more or less than this normal range, cellularity scores were defined as + 1, + 2, + 3 and − 1, − 2, − 3, respectively. In addition, for each 10% increase in hematopoietic precursors following irradiation-induced BM suppression, regeneration scores were defined as 1+, 2+, and 3+. The number of multinucleated cells per each 10 high-power field (HPF) were scored as 1+ (less than 1/10HPF), 2+ (2–5/10HPF), and 3+ (5–10/10HPF).

### Enrichment of BM-LMCs, time-lapse imaging, and video microscopy

After killing the mice by cervical dislocation, bones were isolated under sterile conditions, divided into two parts, and put into adapted tubes for centrifugation at 5000*g* for 30 s for collection of the BM. The cell pellet was suspended in Dulbecco’s modified Eagle’s medium (DMEM) (Bioidea, Iran) containing fetal bovine serum (FBS: 10%) (Gibco, USA), penicillin G (100 U/mL) (Bioidea), and streptomycin (100 mg/mL) (Bioidea), followed by passing through a 100-μm pore size cell strainer (SPL Life Sciences, Korea). The medium that passed through this strainer was poured on a cell strainer with 20 μm pore size (Pluriselect®, Germany). Then, the 20-μm strainer was inverted and washed with culture medium to collect the cells attaching on the strainer, and they were in the range of 20 and 100 μm. The isolated LMCs were followed in the first hours post-isolation for their properties by time-lapse imaging and video microscopy using an inverted microscope (Motic 3XR, Spain) equipped with Moticam 5.0 (Motic, Spain). In order to stain the genome content of the cells and fluorescent microscopy, the cells were fixed by paraformaldehyde 4% (Sigma) for 10 min in cold and then 5 min at room temperature (RT). After three times washing with phosphate-buffered saline (PBS), 4′,6-diamidino-2-phenylindole (DAPI) (Sigma) was added to culture plate at the concentration of 0.1 μg/mL. Fluorescent microscopy was performed by Nikon Eclipse TE2000 (Nikon, Japan).

### DNA content analysis

About 10^6^–10^7^ cells were collected and suspended singly in 5 ml of PBS in a centrifuge tube (SPL). Centrifugation in 200*g* for 6 min was performed. The cells were suspended in 0.5 ml of PBS. The suspended cells were transferred dropwise into a tube containing cold ethanol 70%. Then, the cells were kept in fixative solution for at least 2 h. The ethanol-suspended cells were centrifuged for 5 min at 200*g*. Ethanol was decanted thoroughly, and after that, the cell pellet was suspended in 5 ml of PBS. Centrifugation was performed at 200*g* for 5 min. The cell pellet was suspended in 1 ml of PI (Sigma)/Triton X-100 (Sigma) staining solution with RNase A (Thermo Fisher Scientific, USA). For preparing this solution to 10 ml of 0.1% (v/v) Triton X-100 in PBS, 50 μL of 100 μg/mL DNase-free RNase A and 200 μl of 50 μg/ml PI were added. This solution was prepared freshly.

### Multilineage differentiation

Multilineage differentiation was performed according to our previous study [35]. Briefly, for differentiation into adipocytes and osteocytes, the LMCs at 40 h post-isolation were cultured in adipocytes and osteocytes differentiation medium (Stem Cell Technology, Iran). Every two days, half of the medium was replaced with fresh differentiation medium. After 3 weeks, the cells were fixed with paraformaldehyde 4% and stained with Oil Red O (Sigma) or Alizarin Red staining (Sigma) to detect adipocytes and osteocytes, respectively. For chondrocyte differentiation, 500 LMCs were extracted and centrifuged at 200*g* for 5 min to form a micro-mass. The supernatant was then substituted with fresh chondrocyte differentiation medium (Stem Cell Technology). Differentiation was assessed 3 weeks later by Alcian Blue staining (Bioidea, Iran) of 4-μm-thick sections, followed by counterstaining with hematoxylin (Sigma). The experiments were conducted with three biological replicates each with three technical repeats.

### Immunocytochemistry

Anti-adiponectin antibody (Abcam, USA; ab22554) was used for immunostaining of BM-LMCs with a 1/400 dilution. The wells were firstly washed with PBS and then incubated with 4% of cold paraformaldehyde for 10 followed by 5 min at RT. Triton X-100 0.25% was added for 20 min at RT. After washing with PBS (Gibco), H_2_O_2_ 0.3% was added for 20 min in a dark place at RT followed by two times washing with PBS. The cells were incubated with 5% goat serum (Sigma) for 45 min at RT. After removing goat serum, the first antibody diluted in bovine serum albumin (BSA) (Sigma)/PBS 0.1% was added and the plate was kept at 4 °C overnight. Then, the wells were washed with PBS–Tween 0.1%, 3 × 5 min. After incubation with BSA/PBS 1% for 30 min at RT, the second antibody (Abcam, ab205719, 1/1000) was added to wells for 3 h. Subsequently, the wells were washed with PBS–Tween 0.1%, 3 × 5 min. 3,3'-Diaminobenzidine (DAB) (Sigma, Germany) solution was used as chromogen on plates for 10 min at RT. The wells were washed with PBS, 2 × 5 min. Finally, PBS was added to the wells.

### Immunofluorescence staining

Immunofluorescence staining was performed on cultured LMCs at 24 h after isolation using PE-conjugated anti-CD73 (BD Bioscience, USA, #127205, 1/150), PE-conjugated anti-CD31 (eBioscience, USA, #12-0311-82, 1/150), FITC-conjugated anti-CD68 (BioLegend, #137005, 1/300), and PE-conjugated anti-CD34 (BD Bioscience, #551387, 1/75). Briefly, the cells were washed with PBS and 4% of cold paraformaldehyde was added for 20 min. Then, 5% of goat serum was added for 60 min at RT for blocking, and then, remove without washing. The cells were incubated with the antibodies diluted in BSA/PBS 0.1% for 1–2 h at RT after which the wells were washed with PBS–Tween %0.1, 3 × 5 min. The cells were kept in PBS , and imaging was performed using Nikon Eclipse Ti (Nikon). The ratio of positive cells for each marker was determined by inspecting 100 LMCs.

### Cell transplantation

Whole BM was extracted from the femurs of 6–8-week-old GFP-positive C57BL/6 mice (Received from Tehran University of Medical Sciences). Using a cell strainer, the cells sized between 100 and 20 μm were isolated as described in the previous sections. The isolated cells were suspended in DMEM and plated in cell culture dishes (SPL Life Sciences, South Korea). After 40 h, the multinucleated large-sized adherent cells were selected by a microscope and marked. The parts of the dish not marked for polyploidy were scratched by pipette tips to omit mononucleated cells. After three times washing with PBS, the selected multinucleated cells were detached from the culture plate by a cell scraper (SPL). The cells were pooled in a falcon tube and centrifuged at 200*g* for 5 min. Subsequently, the cell pellet was suspended in DMEM and kept at 4º C for transplantation. NOG immunodeficient mice (Received from Tehran University of Medical Sciences) were myeloablated with Busulfan (CelonLabs, India) at the dose of 30 mg/kg. After 24 h, each mouse was transplanted intraperitoneally with around 50–60 LMCs suspended in 500 μL DMEM. As a positive control, a group of myeloablated NOG mice underwent transplantation of the whole BM of the GFP^+^ mice (i.e., each mouse received the whole marrow of one femur suspended in 500 μL of DMEM). The mice were housed in pathogen-free condition with free access to food and water and 12 hour of daylight. Negative control mice were myeloablated, but not transplanted. After two months, the mice were killed and the presence of GFP^+^ cells was evaluated in blood, spleen, and BM.

### Homing assessment by flow cytometry

The presence of GFP-expressing cells was assessed in blood, BM, and spleen of the transplanted mice. The spleen was detached and passed through a 100-μm mesh and then was washed with DMEM/FBS 10%. The isolated blood, BM, and spleen were treated with 10% of red blood cell (RBC) lysis buffer (Cytomatin Gene, Iran) in distilled water. For each 100 μl of cell suspension, 900 μl of the lysis buffer was used. After 5-min incubation at RT, the cell suspensions were centrifuged at 400*g* for 5 min. The supernatant was omitted, and the cell pellet was resolved in 500 μl of cold PBS. Flow cytometry was carried out using BD FACSCalibur (BD Biosciences, USA). Flowjo software version 10 was used for data analysis.

### Immunohistochemistry

Immunohistochemistry was performed as previously described [36]. Anti-adiponectin antibody (Abcam, ab22554, IHC: 1/400, mouse) and anti-GFP antibody (Santa Cruz, sc-390394, IHC: 1/200) were used in this study. For quantification of immunohistochemistry (IHC) results, ten random areas were captured for each day and the DAB-stained regions were quantified using the IHC Toolbox Plugin of ImageJ software.

### Quantitative PCR

Total RNA was extracted using RNX-plus (CinnaGen, Iran) according to the manufacturer’s instruction. To eliminate DNA contamination, the samples were treated with DNase I (Thermo Fisher, Massachusetts). To validate this procedure, mock controls were also employed. Subsequently, random hexamer-primed cDNA synthesis was performed using the first-strand cDNA synthesis kit (YektaTajhiz, Iran). Genes related to multinucleation were selected from the literature, and specific primers were designed (Additional file 2: Table S2). Quantitative PCR was performed using RealQ Plus 2 × Master Mix Green with high ROXTM (Ampliqon, Denmark) by StepOne machine (ABI, USA). The expression of the genes was normalized by considering *Hprt* and *Tfrc* as reference genes. The results were analyzed using the ΔΔCt method.

### Statistical analysis

The Mann–Whitney U test was used to compare means between the groups. Statistical significance threshold was considered at the level of *p* value < 0.05. The calculations were done using GraphPad Prism 8.0 software. Data are presented as mean ± standard deviation (SD).

## Results

### LMCs transfer genomic content through various mechanisms

**Fig. 1:**
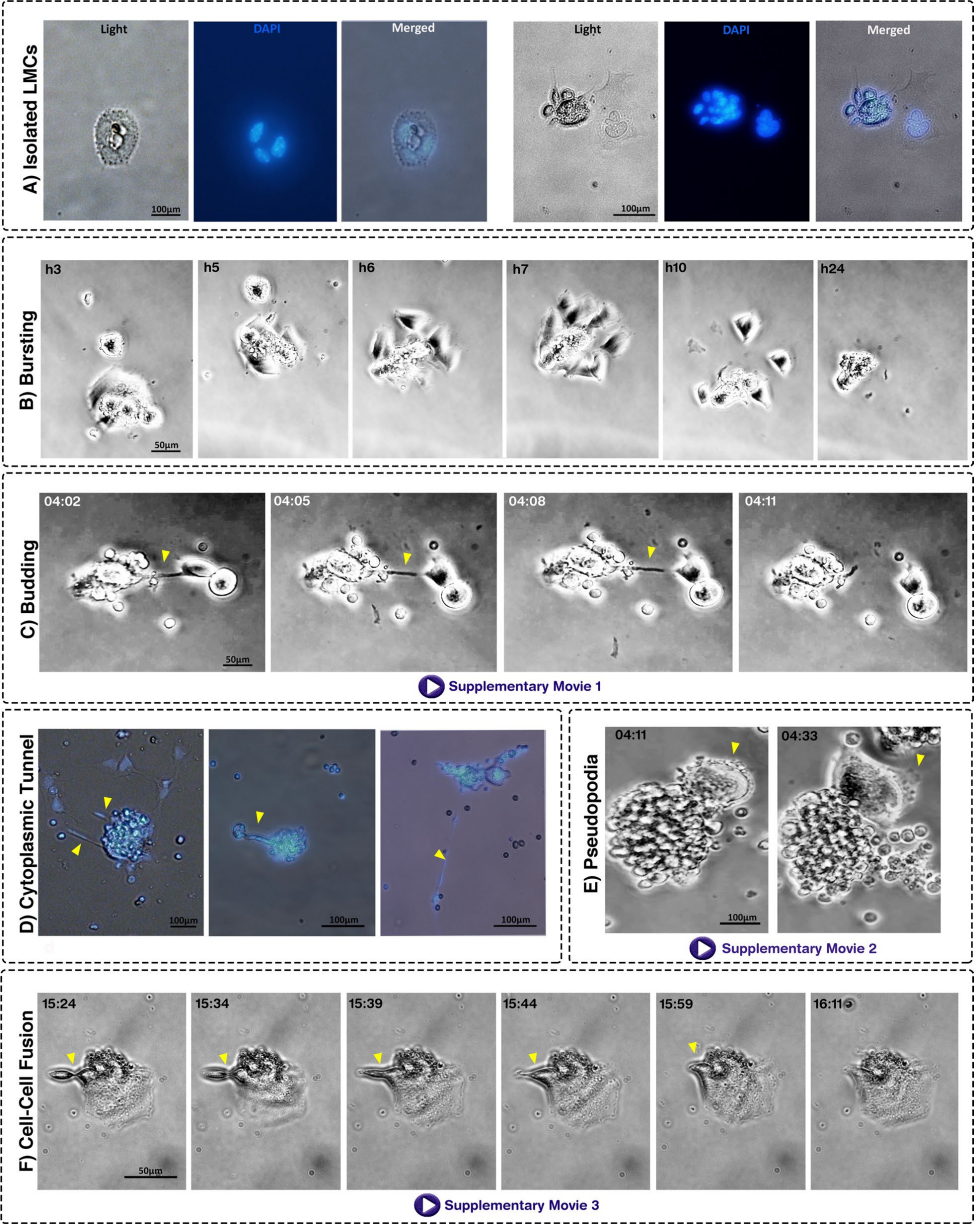
Morphology of LMCs in the culture. **A** Single LMCs isolated by limiting dilution in the first hours post-culture. **B** Bursting of the multinucleated cells into mononucleated cells (time-lapse imaging). **C** Budding of a daughter cell over 10 min. **D** Fluorescent microscopy determined the transfer of genome content to budding progenies. **E** Formation of pseudopodia by the cytoplasm of an LMC. **F** Fusion of mononucleated with a multinucleated giant cell. Times are reported as hour: minute. In each part, the key processes are indicated by arrowheads. Additional observations are available in Additional file 5: Movie S1, Additional file 7: Movie S2, Additional file 8: Movie S3

The frequency of polyploid cells in normal mouse marrow is 3.4 ± 0.3% of all nucleated cells according to cytofluorometric DNA content analysis (Additional file 3: Fig. S1). To isolate these polyploid cells, BM cells sized 20–100 μm were captured using size filtration and cultured at low density in the plate. The selection of this size range is in line with the study of Zhang et al. that reported cancer polyploid cells are three times larger than normal cells [37]. As the average size of BM mononucleated stromal cells is around 17 μm [38], the size of LMCs was assumed to be in the range of 20–100 μm. Notably, 24 h after isolation, the largest diameter of surface-adhered LMCs was 167 ± 35 μm which is compatible with a diameter of 20–100 μm in the suspension (Additional file 4: Fig. S2). Time-lapse imaging was carried out in the primary hours after culturing. Large cells were observed containing multiple nuclei by fluorescent microscopy (Fig. 1A) which are hereafter named large multinucleated cells (LMCs). These cells generated small mononucleated cells through either bursting or budding. In bursting, LMCs rapidly broke down into small pieces resembling a broken plate (Fig. 1B). In budding, however, small cells slowly emerged from the mother cell one by one (Fig. 1C; Additional file 5: Movie S1, Additional file 6: Fig. S3). Interestingly, DAPI staining revealed the cytoplasmic extensions (cytoplasmic tunnels) connecting LMCs to budding progenies harboring nucleic acid (Fig. 1D). Additionally, rapid dynamism of lots of pseudopodia on the border of LMCs was detectable (Fig. 1E; Additional file 7: Movie S2). The cytoplasmic arrangements such as tunnel-like structures and pseudopodia seem to facilitate functions of LMCs. Although the emergence of mononucleated cells from LMCs was detectable, a reverse process was also observed in which one mononucleated cell fused with an LMC (Fig. 1F; Additional file 8: Movie S3).

### BM-LMCs are clonogenic, differentiate into different lineages, and have distinct surface marker and gene expression patterns

**Fig. 2:**
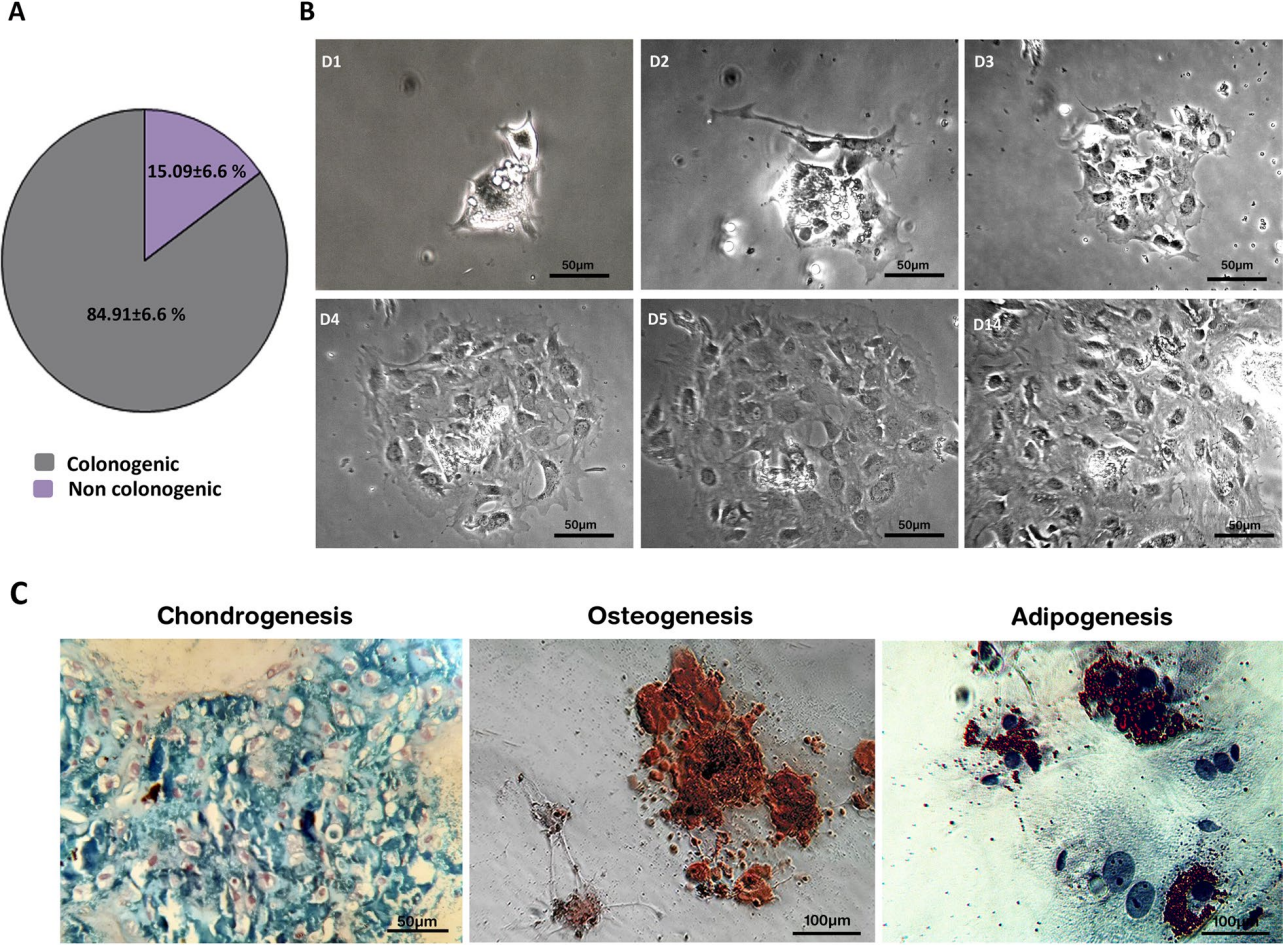
Follow-up of LMCs determined their clonogenic capability and three-lineage differentiation. **A** A considerable fraction of LMCs have the potential of colony formation within a few days after isolation. Data are mean ± SD of three independent experiments. **B** Time-lapse imaging of an LMC in two weeks. **C** The cells had the potential of differentiation to chondrocytes (Alcian Blue-stained), osteocytes (Alizarin Red-stained), and adipocytes (Oil Red-stained)

**Fig. 3:**
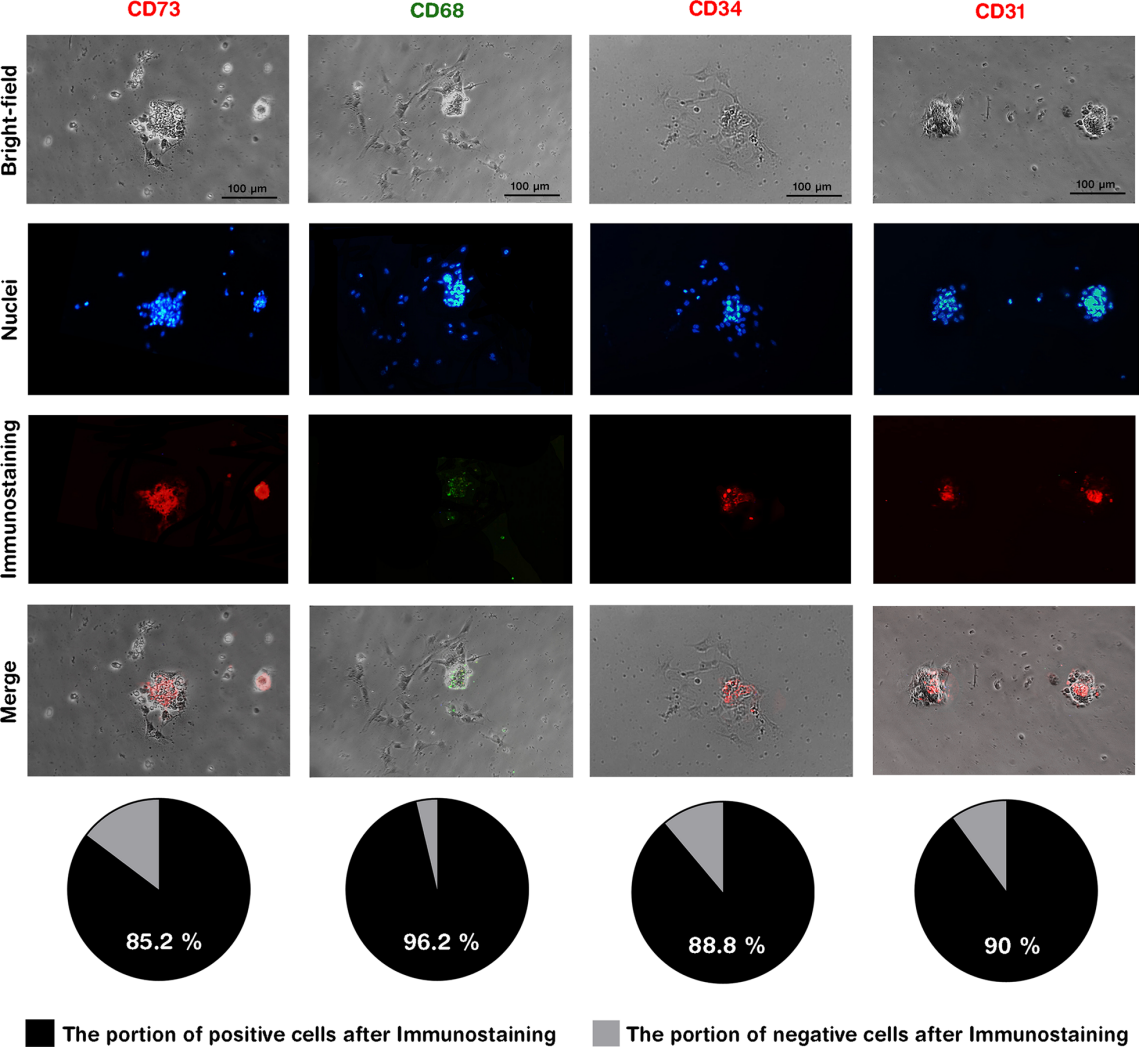
Immunofluorescence staining of LMCs demonstrated the expression of diverse surface markers. Immunostaining of LMCs at 24 h after isolation revealed that these cells express CD34 (as a hematopoietic stem cells marker), CD31 (as endothelial cells marker), CD73 (as one of the mesenchymal stem cells markers), and CD68 (as a macrophage lineage marker) at various intensities. The cells were also incubated with isotype control antibodies as negative controls and remained completely unstained (data shown in Additional file 10: Fig. S5). Scale bars: 100 μm. Pie charts show the percentage of positive and negative cells

The characteristics of LMCs were further investigated by culturing size-filtered cells at low intensity. LMCs were purified by manual elimination of mononucleated cells. The single LMCs were checked for their colony formation capability as well as multi-lineage differentiation. Follow-up of single LMCs in three independent experiments showed that 84.9 ± 6.6% of these cells have the capability of colony formation in primary days after culture (Fig. 2A, B). Furthermore, after 21 days of treatment with differentiation media, progenies of cultured LMCs differentiated into adipogenic, osteogenic, and chondrogenic lineages (Fig. 2C). Notably, the cultured LMCs tended to differentiate into adipocytes even in the absence of adipogenic medium (Additional file 9: Fig. S4). We were also interested to assess the surface markers of LMCs. Hence, 24 h after isolation, the cells were subjected to immunocytochemistry staining with specific antibodies. This assay demonstrated that LMCs are positive for CD34 (as a hematopoietic stem cells marker), CD31 (as an endothelial cell marker), CD73 (as a mesenchymal stem cells marker), and CD68 (as a macrophage lineage marker) as represented in Fig. 3 and Additional file 10: Fig. S5.

Using size filtration, BM-extracted cells sized 20–100 μm and smaller than 20 μm were separately cultured for 24 h, non-adherent cells and debris were removed, and adherent cells were used for gene expression analysis. The expression of genes belonging to cell stemness (*Nanog*, *Oct4*, *Sox2*), cell cycle progression (*Cdk1*, *Chk1*, *E2f1*, *Plk1*), and meiosis (*Sycp3* and *Rec8*) was considerably altered in the LMC-rich fraction compared with the mononucleated cells (Additional file 11: Fig. S6). This finding suggests the involvement of these three processes in multinucleated cells. Further experiments are required to achieve a mechanistic view of the complex gene regulatory circuits in LMCs.

### LMCs are resistant to sub-lethal irradiation and are the key drivers of BM regeneration

**Fig. 4:**
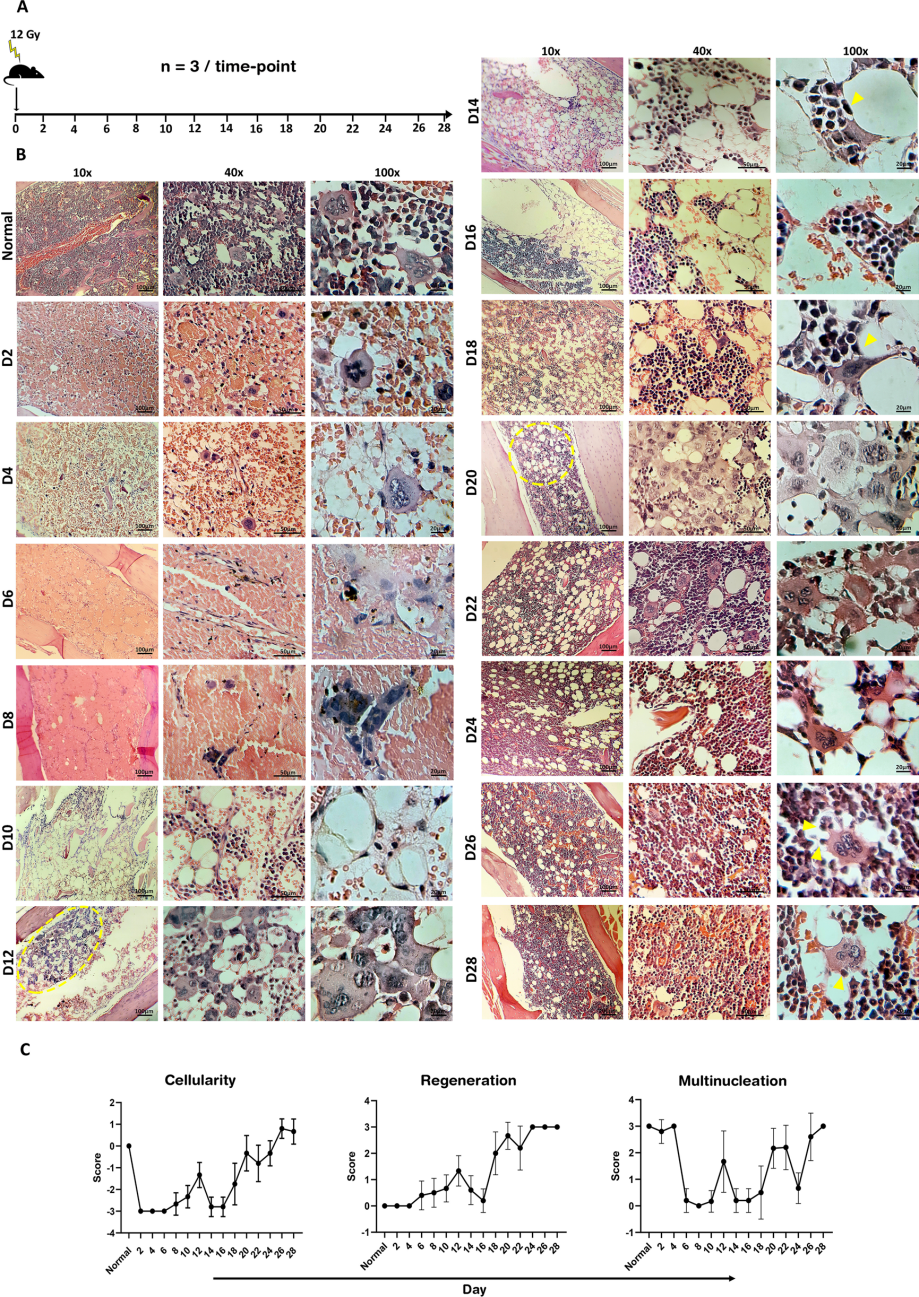
Evaluation of bone marrow regeneration over 28 days post-sub-lethal x-irradiation. **A** Scheme of the experiment design. **B** Tissue sections in different time points by objective lens 10, 40, and 100x (dotted circles: islands of multinucleation, arrowheads: budding and bursting of progenies). Scale bars: left panels: 100 μm, middle panels: 50 μm, right panels 20 μm. **C** Quantitative evaluation of cellularity, regeneration, and multinucleation. The values are represented as mean ± SD

Considering the potential of LMCs to produce mononucleated cells and their high plasticity motivated us to explore their contribution in tissue regeneration. A mouse model of BM ablation was developed by whole-body irradiation (Fig. 4A). After examination of different doses from 8 to 14 Gy, the dose of 12 Gy was selected as it resulted in a reproducible BM destruction and a survival rate above 90%. The femur bones of irradiated mice were harvested for histopathology investigations in two-day time intervals for 28 days. As expected, a vast reduction in cellularity of BM was observed in the primary days post-irradiation which continued with diffused hemorrhages on days 6 and 8 (Fig. 4B). Notably, in the depleted BM, the presence of LMCs which had survived sub-lethal irradiation was prominent since day 2. Regeneration was prominently started at days 10–12 with distinct hyper-cellular regions. These regions were enriched by LMCs (Additional file 12: Fig. S7) which serve as sources of new mononucleated cells (Fig. 4B day 12, dotted circle). BM cellularity increased in the consecutive days within prominent regions in which LMCs broke down into mononucleated cells forming puzzle-like shapes (Fig. 4B days 14 and 18; arrowhead). On day 20, cellularity was similar to normal BM (Fig. 4C), but LMC-rich areas were still detectable (Fig. 4B, day 20 dotted circle, Additional file 12: Fig. S7). On days 26 and 28, BM was hyper-cellular (Fig. 4C) with observable LMCs which generate mononucleated cells through budding (Fig. 4B days 26 and 28, arrowheads).

**Fig. 5:**
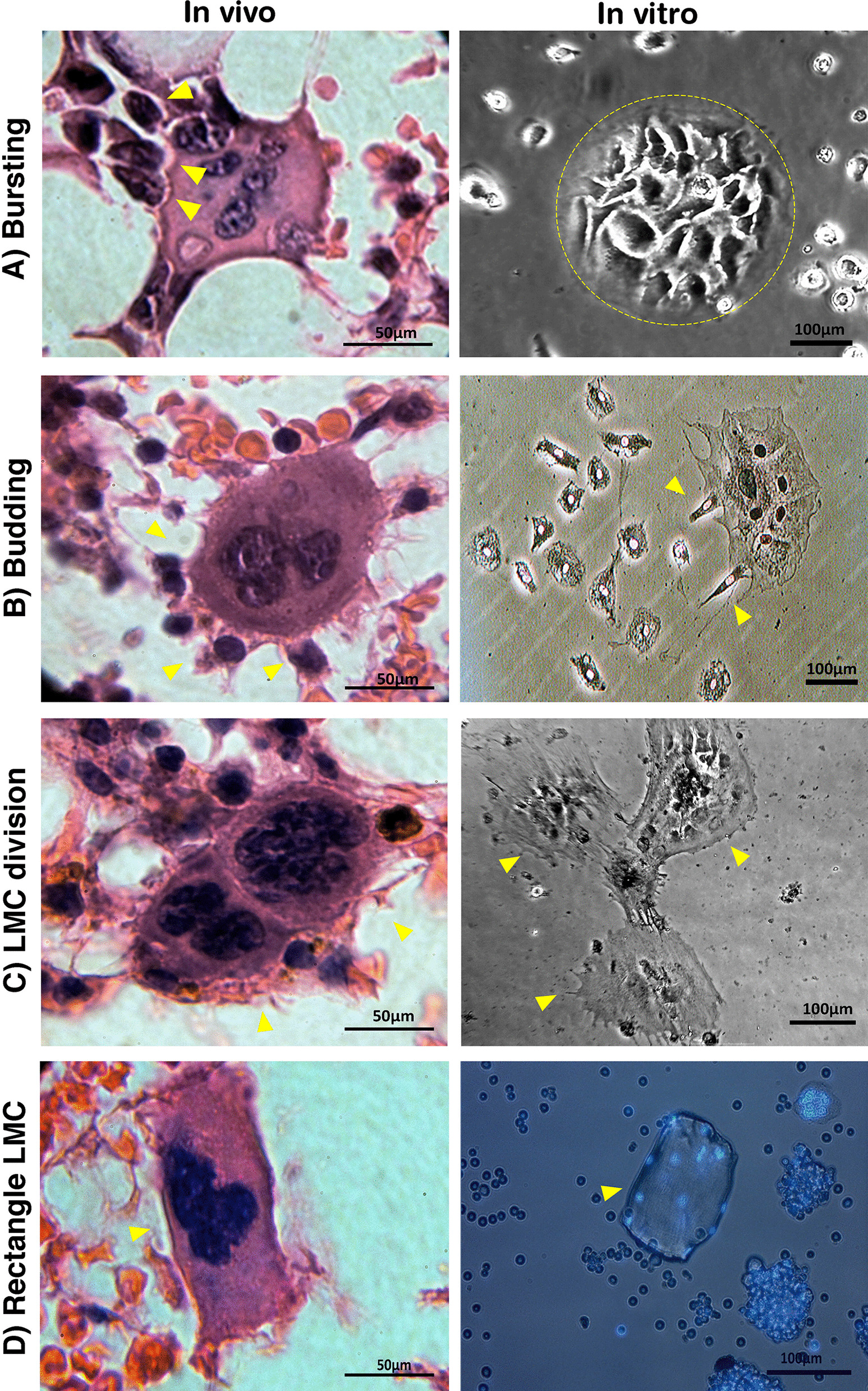
In vivo and in vitro analogy of LMCs features. **A** Bursting of an LMC into daughter cells. **B** Budding of mononucleated progenies from a multinucleated cell. **C** Division of a multinucleated cell into two multinucleated daughter cells. **D** Rectangle-shaped multinucleated cell. Scale bars: left panels: 50 μm, right panels: 100 μm. The key processes are indicated by arrowheads and the dotted-circle

Culture of BM in parallel to histopathology allowed us to overlay in vitro and in vivo morphologic characteristics of the LMCs. The bursting of LMCs in tissue sections was detectable in cultured BM cells on days 14–28 (Fig. 5A). The other prominent observation was the budding of progenies from LMCs in both sections and cultured cells (Fig. 5B). Furthermore, the split of an LMC into two new LMCs was repeatedly observed in both conditions (Fig. 5C). Although most of the LMCs had rounded or irregular shapes, rectangular cells were occasionally observed suggesting the presence of cells with specific functions in BM whose role remained to be understood (Fig. 5D). These observations are in agreement with our hypothesis that BM multinucleated cells are the sources of rapid regeneration by producing BM mononucleated cells through different mechanisms.

### Multinucleated adipocytes and fat droplets are essential elements in BM regeneration

**Fig. 6:**
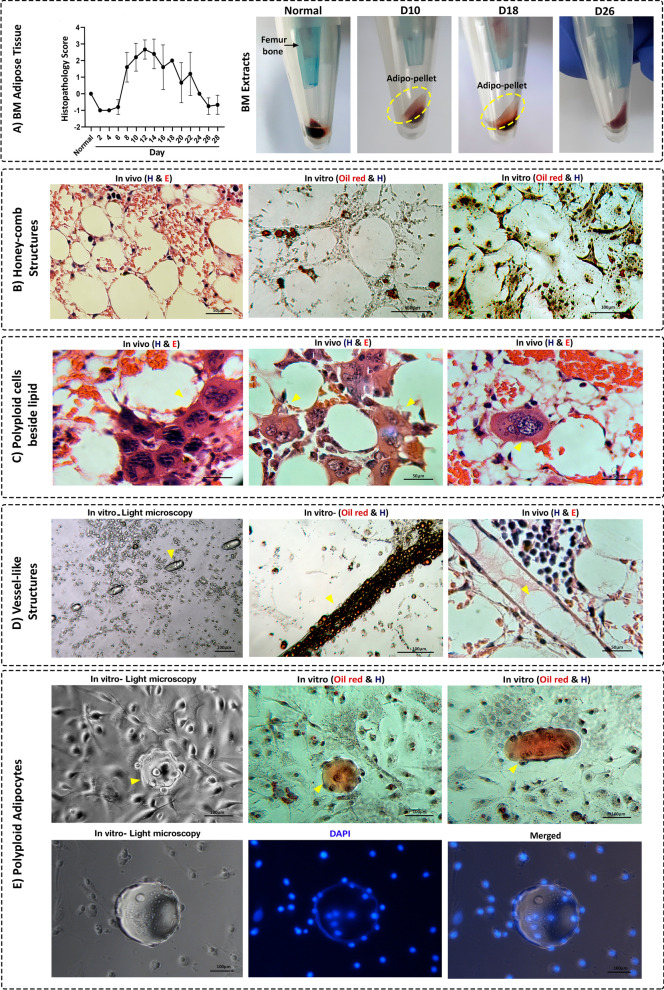
Adipocytes as critical elements of tissue regeneration. **A** Over 28 days post-sub-lethal X-irradiation, the BM sections were inspected and scored for the adipocyte content. The scores are demonstrated in the curve as mean ± SD. Also, a substantial fraction of BM cell pellet constituted of adipocytes during the regenerative process. **B** Honey-comb structures formed by fat cells. Representative image from H&E section of BM at day16 post-irradiation (left). Culture of ablated BM (day16) after 2 weeks (middle) and 4 weeks (right). **C** Multinucleated cells were observed around the structures generated by fat cells in the BM sections of the sub-lethally irradiated mice on day 16 post-irradiation. **D** Adipocytes formed vessel-like structures. Adipocytes lined up immediately after culture (left). Oil Red staining of oriented adipocytes of ablated BM (day 18) 5 weeks after culture (middle). In the BM (day 20), the newly formed vessels contained remnants of adipocytes (left). **E** Multinucleation of adipocytes. Multinucleated adipocytes were observed by optical microscopy (left) and after Oil Red staining (middle). Another representative is shown in right. Multinucleation of adipocytes was furtherly observed by DAPI staining (lower panels). Representative phenomena are shown by arrowheads

In line with previous studies [39], follow-up of BM cultures demonstrated a prominent increment of adipocytes in the first week after irradiation that was replaced by marrow hematopoietic and stromal cells in the consecutive days (Figs. 4B and 6A). Although the presence of a high percentage of adipocytes in the BM is classically related to inefficiency of hematopoiesis and result of aging [40], the active presence of adipocytes in BM regeneration suggests their fundamental role in this process. It has been shown that adipocytes are energy reservoirs accelerating highly metabolic phenomena [41]. Regarding the size and 3D structure of adipocytes, it is reasonable to assume that these cells could act as potential physical backbones for the reconstitution of depleted cells. In line with this hypothesis, the fat cells could form honeycomb structures both in vitro and in vivo (Fig. 6B). Also, multinucleated cells were mostly found around these structures (Fig. 6C) which recapitulates the relation of adipocytes and multinucleated cells to tissue regeneration. On the other hand, adipocytes formed linear structures in the culture of irradiate BM which, in the long term, shaped 3D vessel-like structures (Fig. 6D). Accordingly, remnants of adipocytes were present in the newly formed vessel-like structures post-BM ablation (Fig. 6D). These findings raise the hypothesis that adipocytes are the primary backbones of newly formed vessels, which requires further validation in future studies.

We also observed multinucleated adipocytes in the culture of ablated BM. The multinucleated adipocytes share similar features with LMCs as they budded progenies or transferred genomic content through the cytoplasmic tunnel (Fig. 6E). Based on the above-mentioned findings, adipocytes can be regarded as critical components of BM regeneration and the presence of multinucleated adipocytes is necessary for the promotion of regeneration in the BM.

### Multinucleated adiponectin-expressing cells are involved in tissue regeneration

Augmentation of the number of adipocytes during BM regeneration as well as their multinucleation motivated us to further assess the contribution of this lineage to tissue repair. There is a bundle of evidence highlighting that BM adipocytes are the promoters of tissue regeneration. Morrison and colleagues determined that perivascular adipocytes expressing adiponectin are frontiers in tissue regeneration and the main source of stem cell factor (SCF) [42–44]. Furthermore, they demonstrated that the SCF produced by adiponectin-positive adipocytes is urgent for the maintenance of hematopoiesis in the ablated bone [45].

**Fig. 7:**
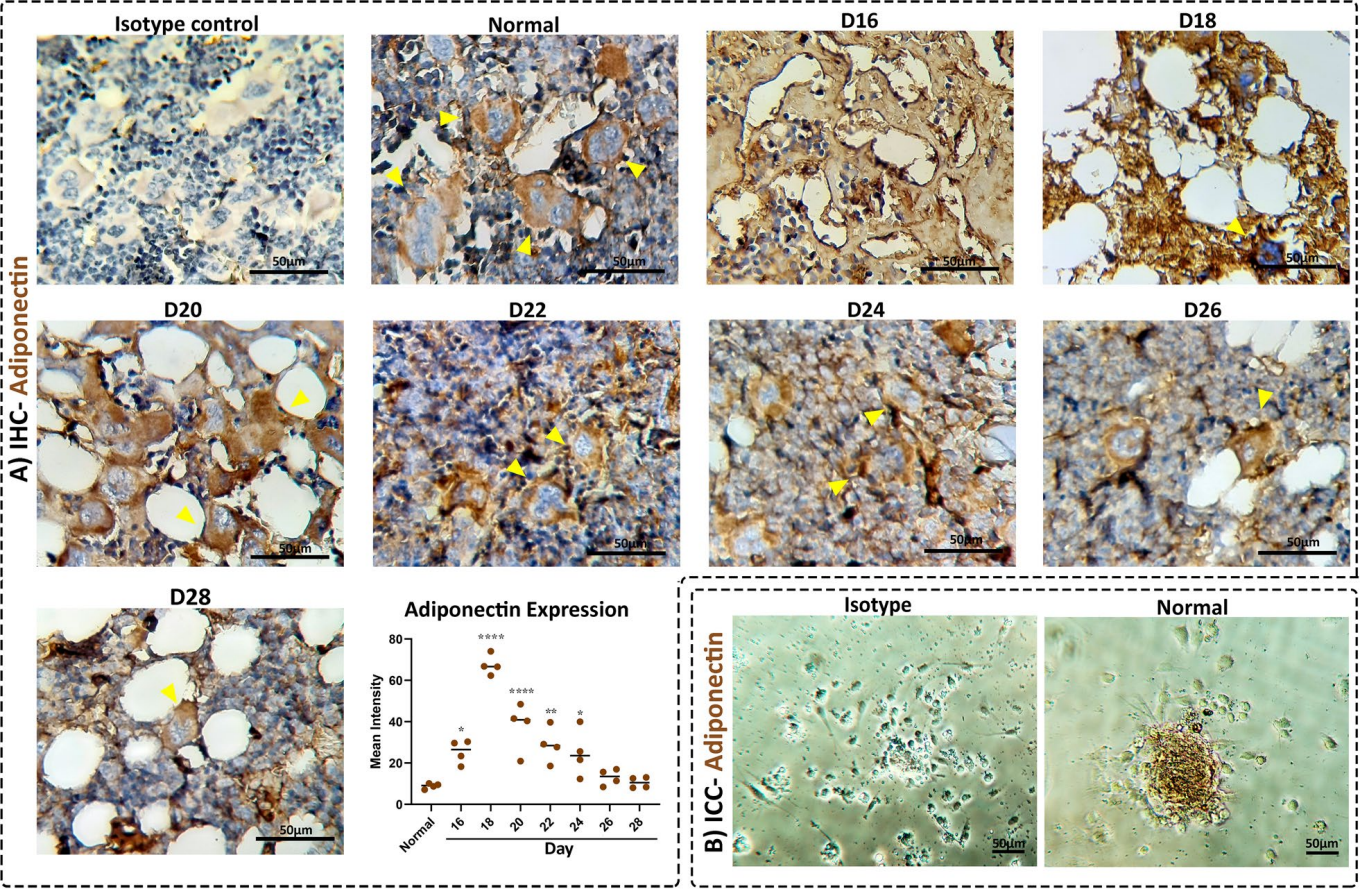
Expression of adiponectin by multinucleated cells. **A** Immunohistochemistry for adiponectin in BM of myeloablated mice over 28 days. The arrowheads determine multinucleated adiponectin-positive adipocytes. Scale bar: 50 μm. *p value < 0.05, **p value < 0.005, and ****p value < 0.0001. **B** BM of the normal mouse was isolated; one-day post-culture expression of adiponectin was assessed by immunocytochemistry. Scale bar: 50 μm

For assessment of the function of adiponectin-positive cells during BM regeneration, immuno-phenotyping was performed on tissue sections as well as cultured BM. Interestingly, a considerable number of adiponectin-positive cells were multinucleated in the normal BM. From day 16 to 20 post-BM ablation, the number of adiponectin-positive cells dramatically increased, including not only multinucleated cells but also mononucleated progenies surrounding them (Fig. 7A). From day 22, the adiponectin-positive cells gradually decreased. Immunocytochemistry on the culture of BM 24 h post-extraction determined that multinucleated cells express adiponectin in their cytoplasm, despite the negativity of mononucleated progenies for this marker which further confirmed the in vivo findings (Fig. 7B). These findings suggest that multinucleated regenerative cells are adiponectin positive. The above descriptions on the rapid emergence of adipocytes in the middle of the BM regeneration course besides the findings on the expression of adiponectin by multinucleated cells highlighted the role of adipocyte lineage in tissue regeneration.

### BM-LMCs could repopulate both hematopoietic and stromal lineages upon transplantation into injured mice

**Fig. 8:**
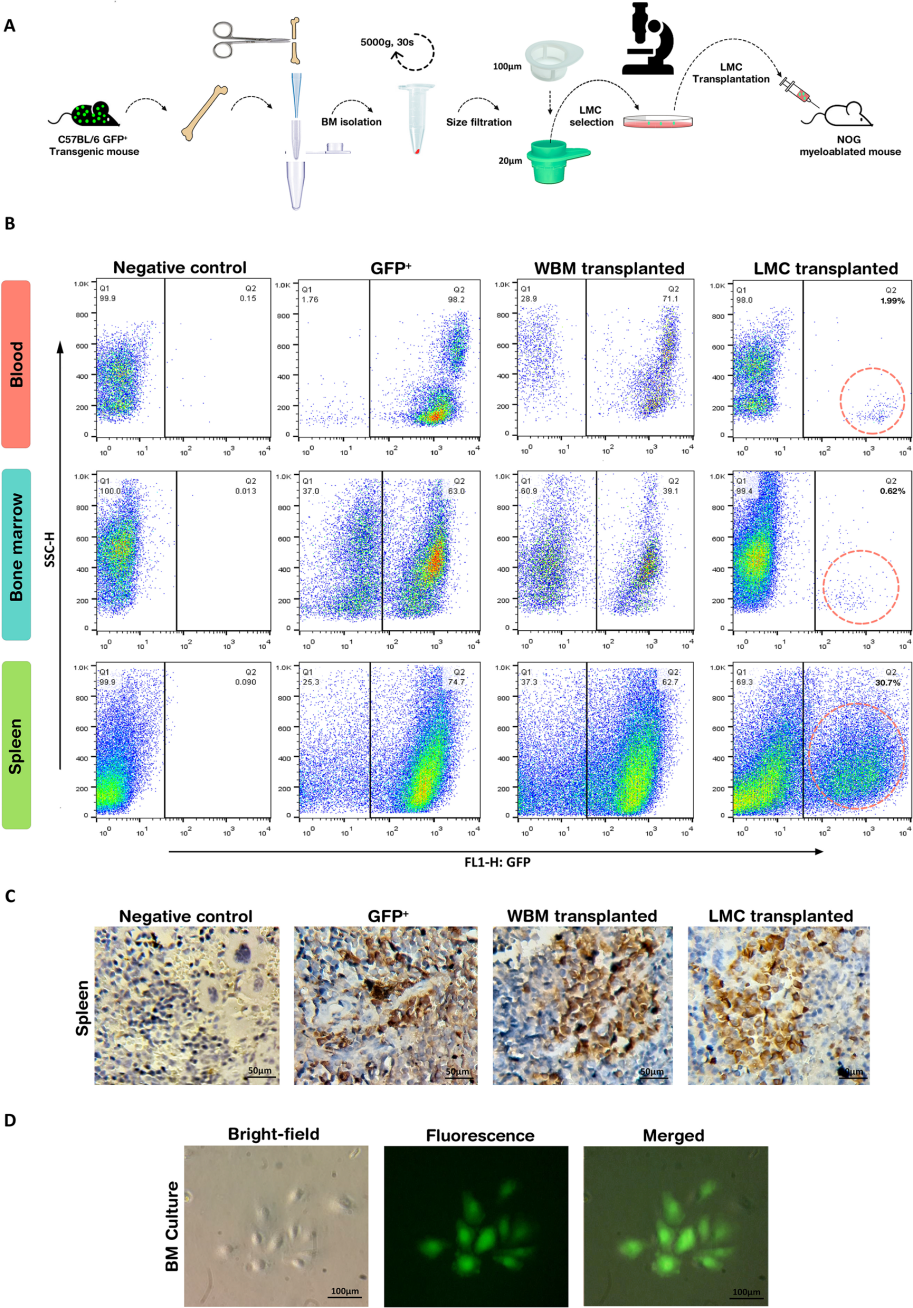
Transplanted LMCs reconstitute hematopoietic and stromal lineages in myeloablated NOG mice. **A** Procedure of isolation and transplantation of BM-LMCs. **B** Flow cytometry of peripheral blood, BM, and spleen for GFP^+^ cells in normal, GFP transgenic, whole BM-transplanted, and LMC-transplanted mice. Dotted circles show the population of GFP-positive cells. **C** Immunohistochemistry of spleen sections for GFP. Scale bar: 50 μm. **D** Colonies of GFP^+^ cells two weeks after culturing BM of LMC-transplanted mice. *WBM* whole bone marrow, *LMC* large multinucleated cell. Scale bar: 100 μm

An increment in LMCs during BM regeneration and their potential in producing mononucleated progenies in vitro motivated the authors to further assess the capability of LMCs in reconstituting tissues in vivo. In this regard, BM-LMCs harvested from GFP transgenic mice were transplanted to myeloablated NOG mice and followed up to determine their regenerative capacity (Fig. 8A). In parallel, the mice receiving whole BM or culture medium were employed as positive and negative controls, respectively.

Notably, progenies of transplanted GFP^+^ LMCs were detectable as distinct populations in peripheral blood, spleen, and BM after two months. Interestingly, the LMC-derived cells constituted around 30% of the recipient spleen cells (Fig. 8B). Immunohistochemistry further confirmed this unexpected observation (Fig. 8C). BM of LMC-transplanted mice was also cultured. After two weeks, colonies of GFP^+^ cells were observable that resembled colony-forming unit fibroblast (Fig. 8D). These observations demonstrated the ability of LMCs to produce stromal cells in addition to the reconstitution of hematopoietic lineages.

## Discussion

This study investigated the contribution of multinucleated cells in BM regeneration. Time-lapse follow-up of the sub-lethally irradiated mice determined the high resistance of polyploid cells to injury as well as their role as regeneration promoters. Polyploid cells survived while the BM was depleted by 95% the first days after irradiation. The stress tolerance of the polyploid phenotype has been reported in different organisms. Yeasts become polyploid in response to hyperosmotic, metal, and starvation stresses [6–8] to augment gene expression capacity and compensate for the loss of function mutations [9]. In mammals, hepatocytes took the advantage of polyploidization when face with stressors such as aging, hepatectomy, iron-overload, and toxins [46–48]. In this study, the formation of LMCs islands was observed in the sites of regeneration after BM ablation. Parallel in vitro and in vivo evidence determined rapid production of mononucleated progenies by LMCs through budding or bursting which is also demonstrated in polyploid giant cancer cells [49]. In our recent review, it was discussed that the rapid releasing of mononucleated cells from polyploid cells provides a strategic advantage for adaptation with stress conditions in both regeneration and cancer [29].

In contrast to general descriptions introducing BM multinucleated cells as megakaryocytes, transplantation and multi-lineage differentiation assays of this study determined that these cells are more potent than a differentiated cell (megakaryocyte) and seem to belong to upper levels of cell stemness hierarchy. Furthermore, in vitro three-lineage differentiation of LMCs and generation of both stromal and hematopoietic cells from transplanted LMCs suggested a single origin for these cells and scrutinized current belief on the disparity of the origin of stromal and hematopoietic lineages. In agreement, Krause et al. determined that a single BM-derived cell has both hematopoietic and non-hematopoietic differentiation capability and engraftment into the epithelium of other organs [50]. Moreover, a study by Dominici et al. showed that both hematopoietic cells and osteoblasts could be derived from a common primitive non-adherent cell type [51]. Multiple investigations demonstrated that hematopoietic stem cell (HSC) colonies are the origin of BM stromal cells [52–54] and vice versa [55]. The observations on the plasticity of LMCs in this study are analogous to previous reports on the differentiation capacity of PGCCs [37, 56].

A key finding of this study is the role of adipocytes in tissue regeneration. Adipocytes dramatically increased in the second and third weeks after irradiation correlated with the progression of tissue regeneration, suggesting their fundamental role in tissue repair. Although some studies mentioned adipocytes as negative regulators of hematopoiesis [57] and a consequence of aging and some metabolic disorders [40, 58], this study proposes the possible role of adipocytes in regeneration. It was previously shown that marrow adipocytes tightly correlate with tissue metabolism and supplementation of ATP [59]. Furthermore, marrow adipose tissue is a source of various cytokines such as adiponectin [60], leptin [61], RANKL [62], and DPP4 [63]. Another important but largely ignored issue about adipocytes is their ability to form specific structures that potentially provide mechanical support for regenerating cells. Our in vitro and in vivo observations determined that adipocytes form honeycomb scaffolds. Furthermore, they could orientate in linear structures and facilitate the formation of primary vessels. Although lining up of macrophages on the culture dish has been previously reported by Levy et al. in 1976 [64], to the best of our knowledge, orientation of adipocytes has not been reported before. Further studies are required to unravel the molecular mechanisms behind the formation of these well-organized structures and their functional significance.

Our separate findings on the role of multinucleated cells and adipocytes on regeneration came together with the observation of polyploid adipocytes that are capable of budding progenies in the culture of myeloablated mice marrow. Furthermore, we noticed the tendency of LMCs for differentiation to adipocytes even in the absence of exogenous adipogenic stimulation, suggesting the potential for the activation of gene regulation circuits governing adipocytes. This high capacity of LMCs to differentiate to adipocytes raises concerns regarding their mal-differentiation for regenerative applications that should be addressed in future studies. Also, Immune-phenotyping assays determined that a subset of BM multinucleated cells is positive for adiponectin. According to the study of Zhou et al., BM adiponectin-positive cells, which predominantly increase upon injury, not only produce a considerable fraction of stem cell factor needed for hematopoiesis but also are the origin of most of the mesenchymal stem cell in the marrow [44]. Moreover, Zhang et al. showed that in transgenic mice lacking the stem cell factor originated from adiponectin-positive cells, hematopoietic stem/progenitor cells decrease drastically even in the steady states [65]. These reports underscore the critical role of BM adipose tissue in hemostasis of BM hematopoiesis and maintenance of stem cell niches [66]. Putting together the pieces of BM regeneration puzzle, it is contemplated that polyploidy of adiponectin-positive cells augments their capacity for advancement of regeneration of BM not only as structural scaffolds and suppliers of energy, but also as the source of newly formed progenies upon BM ablation. These findings open up new horizons in the field of regenerative medicine with critical role of polyploid adipocytes.

## Conclusion

Taken together, the results of this study underscore the role of LMCs in marrow tissue regeneration. Moreover, the provided evidence on the contribution of marrow adipose tissue in BM reconstitution indicates the necessity of further detailed investigations. This study raises multiple questions on the molecular mechanisms and metabolic pathways of LMCs, the origin and identity of these cells in the hierarchy of stem cells, and the mechanisms of adipocytes involvement in tissue regeneration.

## Supplementary Information

**Additional file 1. Table S1**: Histopathology scoring of irradiated mice BM sections.

**Additional file 2. Table S2**: List of primers.

**Additional file 3. Fig. S1**: The frequency of polyploid cells in normal BM. A. A representative graph for cytofluorometric DNA content analysis of BM. B. The frequency of different BM populations is quantified based on three independent experiments.

**Additional file 4. Fig. S2**: Average size of BM-LMCs **A**. Size determination of BM-isolated LMCs. Values are mean ± SD. **B**. A representative image of a cultured LMC at 24 h after isolation.

**Additional file 5. Movie S1**: Cell budding. Through budding, small daughter cells slowly emerge from the mother cell one by one.

**Additional file 6. Fig. S3**: Time-lapse imaging of budding cells. In the top panel, the derivation of a mononucleated cell from an LMC is shown during 10 h. The bottom panel represents the occasional dividing of an LMC into polyploid progenies. Scale bar: 100 µm.

**Additional file 7. Movie S2**: Pseudopodia formation. The rapid movement of lots of pseudopodia on the border of LMCs was observed.

**Additional file 8. Movie S3**: Cell–cell fusion. Although the emergence of mononucleated cells from LMCs was detectable, a reverse process was also observed in which one mononucleated cell fuses with an LMC.

**Additional file 9. Fig. S4**: Spontaneous differentiation of LMCs into adipocytes. Even in the absence of an adipogenic induction, LMCs could differentiate to adipocytes as revealed by Oil Red O staining of lipid vacuoles. The cells are counterstained with hematoxylin in the right panel. Scale bar: 100 µm.

**Additional file 10. Fig. S5**: Negative controls for the immunocytochemistry assay. LMCs stained with the isotype control antibodies were completely negative for the fluorescence signals. This observation validates the immunocytochemistry data demonstrated in Fig. 3.

**Additional file 11. Fig. S6**: The expression of genes related to multinucleation. The bone marrow extracts were passed through a 100-µm pore size filter and then through a 20-µm filter. The cells that attached to the surface of the 20-µm mesh and those that passed through it were separately cultured for 24 h. Non-adherent cells and debris were washed, and the attached cells were used for RNA isolation and cDNA synthesis. Quantitative gene expression analysis on genes related to multinucleation demonstrated that the LMC-rich 20–100 µm fraction has substantially higher expression of *Sox2*, *Cdk1*, *Chk1*, and *E2f1* compared to the other population that is rich of mononucleated cells. On the other hand, the genes such as *Plk1*, *Nanog*, *Oct4*, *Sycp3*, and *Rec8* are down-regulated in multinucleated cells.

**Additional file 12. Fig. S7**: Islands of LMCs during BM regeneration. In days 12 and 20 post-sub-lethal irradiation, LMC-rich foci are observed in BM sections.

## Data Availability

All data generated or analyzed during this study are included in this published article and its supplementary information files.

## Abbreviations

BM: Bone marrow
BSA: Bovine serum albumin
DAB: 3,3′-Diaminobenzidine
DAPI: 4′,6-Diamidino-2-phenylindole
DMEM: Dulbecco’s modified Eagle’s medium
FBS: Fetal bovine serum
GFP: Green fluorescent protein
HSC: Hematopoietic stem cell
IHC: Immunohistochemistry
LMCs: Large multinucleated cells
MSCs: Mesenchymal stromal cells
PBS: Phosphate-buffered saline
PGCCs: Polyploid giant cancer cells
RBC: Red blood cell
RT: Room temperature
SCF: Stem cell factor
