# Supplementary material for: Regenerative potential of multinucleated cells: bone marrow adiponectin-positive multinucleated cells take the lead
Source: Stem Cell Res Ther. 2023 Jul 4;14:173. doi: 10.1186/s13287-023-03400-w (PMC10320956; doi:10.1186/s13287-023-03400-w)
Supplement: Supplementary file 1 — Additional file 1. Table S1: Histopathology scoring of irradiated mice BM sections. [file 13287_2023_3400_MOESM1_ESM.pdf]

Supplementary Table 1: Histopathology scoring of irradiated mice BM sections

	**3+**	**2+**	**1+**	**0**	**1-**	**2-**	**3-**
**Cellularity**	> 90%	80-90%	70-80%	30-70%	20-30%	10-20%	< 10%
**Adipose tissue**	> 90%	80-90%	70-80%	30-70%	20-30%	-	-
**Multinucleation**	5-10 multinucleated cells/10 HPF	2-5 multinucleated cells /10 HPF	1 multinucleated cells /10 HPF	None	-	-	-
**Regeneration**	> 90%	80-90%	70-80%	None	-	-	-
