# Supplementary material for: Regenerative potential of multinucleated cells: bone marrow adiponectin-positive multinucleated cells take the lead
Source: Stem Cell Res Ther. 2023 Jul 4;14:173. doi: 10.1186/s13287-023-03400-w (PMC10320956; doi:10.1186/s13287-023-03400-w)
Supplement: Supplementary file 2 — Additional file 2. Table S2: List of primers. [file 13287_2023_3400_MOESM2_ESM.pdf]

Additional file 2: Supplementary table 2: List of primers

Gene name	Forward primer	Reverse primer
**Oct4**	CGTTCTCTTTGGAAAGGTGTT	ATCTCCTGAAGGTTCTCATTGT
**Nanog**	AACTCTCCTCCATTCTGAACC	GTGCTGAGCCCTTCTGAAT
**Sox2**	AGGAAAGGGTTCTTGCTGGG	GACCACGAAAACGGTCTTGC
**Plk1**	GGTGGACTATTCGGACAAGTA	TTGAGGAGAGTGATCTTCTTCAT
**Chk1**	TTGATAGAATTGAGCCAGACATAG	GCCAAGCCAAAGTCAGAGA
**Sycp3**	ATGATGGAAACTCAGCAGCA	GCATGCCTCTTAGCTAATGTTTT
**Rec8**	CAGAACCCCAACTCTCTCTGG	GAGCATTCTTTGGGGGACCT
**E2f1**	GTGGATTCTTCAGAGACATTTCA	GTGGAGGGAGGTGATGGT
**Cdk1**	CAGAGAGGGTCCGTCGTA	ACCGTAAGTACCTTCTCCAATT
**HPRT**	CGTCGTGATTAGCGATGATG	AGTCTTTCAGTCCTGTCCATAA
**TFRC**	TGCATTGCGGACTGTAGAG	CCCACCAAACAAGTTAGAGAAT
