# Supplementary material for: Regenerative potential of multinucleated cells: bone marrow adiponectin-positive multinucleated cells take the lead
Source: Stem Cell Res Ther. 2023 Jul 4;14:173. doi: 10.1186/s13287-023-03400-w (PMC10320956; doi:10.1186/s13287-023-03400-w)
Supplement: Supplementary file 3 — Additional file 3. Fig. S1: The frequency of polyploid cells in normal BM. A. A representative graph for cytofluorometric DNA content analysis of BM. B. The frequency of different BM populations is quantified based on three independent experiments. [file 13287_2023_3400_MOESM3_ESM.pdf]

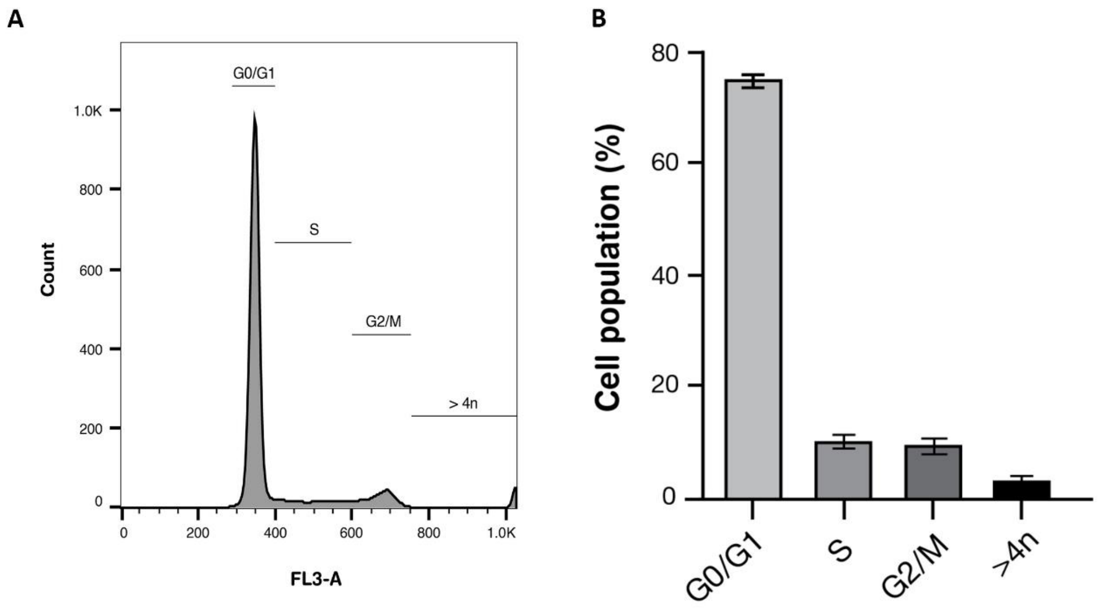

Supplementary Figure 1: The frequency of polyploid cells in normal BM. A. a representative graph for cytofluorometric DNA content analysis of BM. B. The frequency of different BM populations is quantified based on three independent experiments (Values are shown as mean±SD).
