# Supplementary material for: Regenerative potential of multinucleated cells: bone marrow adiponectin-positive multinucleated cells take the lead
Source: Stem Cell Res Ther. 2023 Jul 4;14:173. doi: 10.1186/s13287-023-03400-w (PMC10320956; doi:10.1186/s13287-023-03400-w)
Supplement: Supplementary file 4 — Additional file 4. Fig. S2: Average size of BM-LMCs A. Size determination of BM-isolated LMCs. Values are mean ± SD. B. A representative image of a cultured LMC at 24 h after isolation. [file 13287_2023_3400_MOESM4_ESM.pdf]

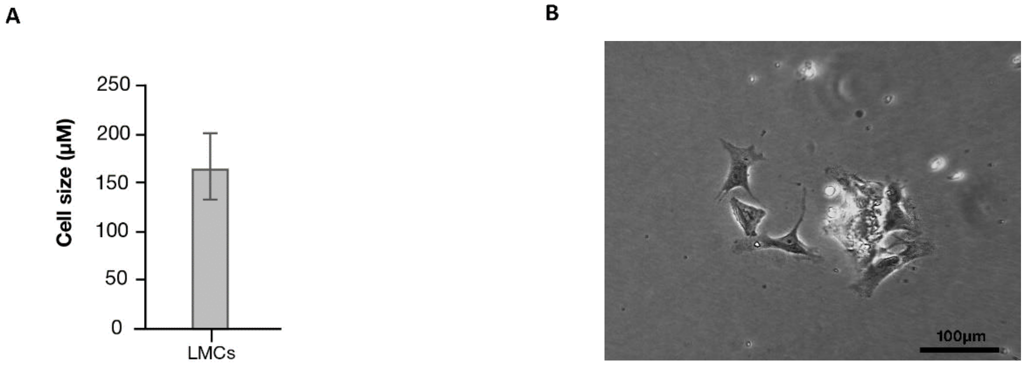

Supplementary Figure 2: Average size of BM-LMCs. A. Size determination of BM isolated LMCs. Values are mean ± SD. B. A Representative image of a cultured LMC at 24 hours after isolation.
