# Supplementary material for: Regenerative potential of multinucleated cells: bone marrow adiponectin-positive multinucleated cells take the lead
Source: Stem Cell Res Ther. 2023 Jul 4;14:173. doi: 10.1186/s13287-023-03400-w (PMC10320956; doi:10.1186/s13287-023-03400-w)
Supplement: Supplementary file 6 — Additional file 6. Fig. S3: Time-lapse imaging of budding cells. In the top panel, the derivation of a mononucleated cell from an LMC is shown during 10 h. The bottom panel represents the occasional dividing of an LMC into polyploid progenies. Scale bar: 100 µm. [file 13287_2023_3400_MOESM6_ESM.pdf]

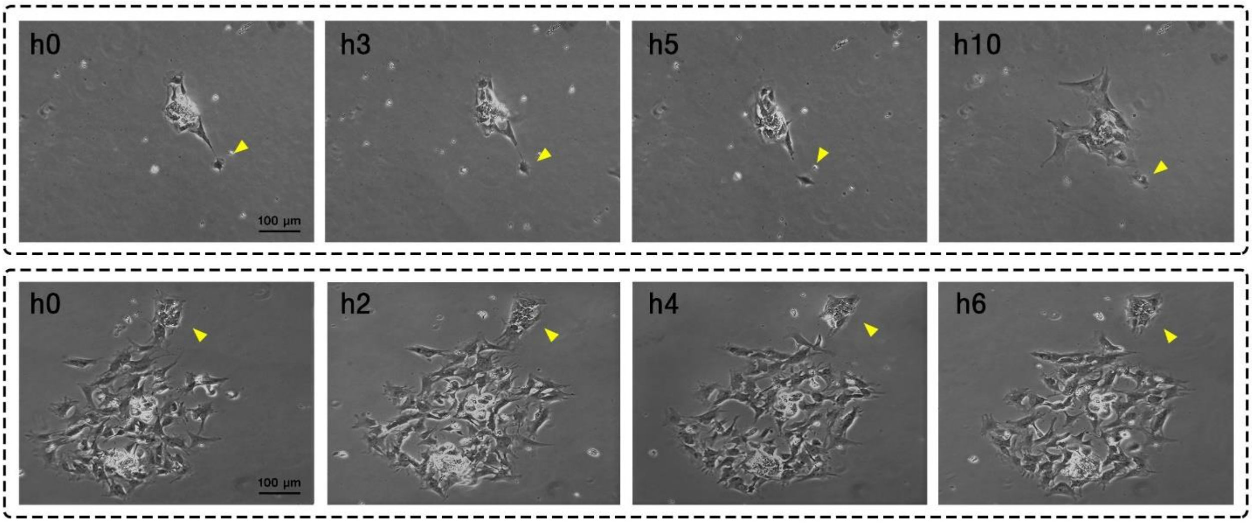

Supplementary Figure 3: Time-lapse imaging of budding cells. In the top panel, the derivation of a mononucleated cell from an LMC is shown during 10 hours. The bottom panel represents the occasional dividing of an LMC into polyploid progenies. Scale bar: 100μm
