# Supplementary material for: Regenerative potential of multinucleated cells: bone marrow adiponectin-positive multinucleated cells take the lead
Source: Stem Cell Res Ther. 2023 Jul 4;14:173. doi: 10.1186/s13287-023-03400-w (PMC10320956; doi:10.1186/s13287-023-03400-w)
Supplement: Supplementary file 9 — Additional file 9. Fig. S4: Spontaneous differentiation of LMCs into adipocytes. Even in the absence of an adipogenic induction, LMCs could differentiate to adipocytes as revealed by Oil Red O staining of lipid vacuoles. The cells are counterstained with hematoxylin in the right panel. Scale bar: 100 µm. [file 13287_2023_3400_MOESM9_ESM.pdf]

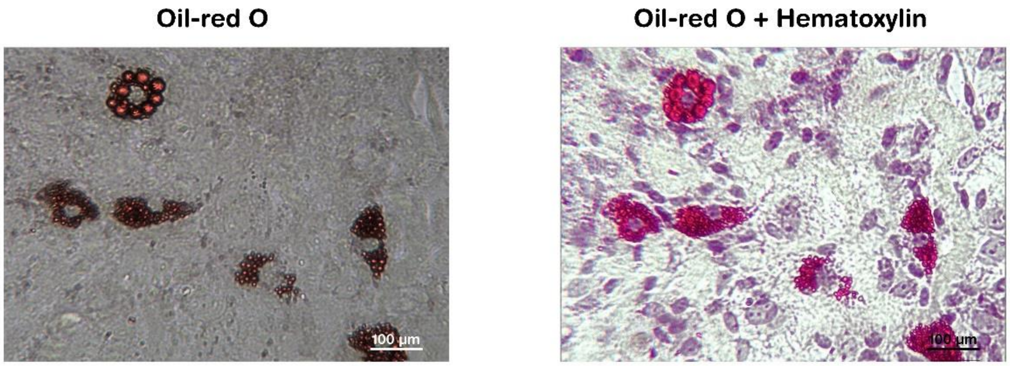

Supplementary Figure 4: Spontaneous differentiation of LMCs into adipocytes. Even in the absence of adipogenic induction, LMCs could differentiate to adipocytes as revealed by Oil Red O staining of lipid vacuoles. The cells are counterstained with hematoxylin in the right panel. Scale bar: 100 µm
