# Supplementary material for: Regenerative potential of multinucleated cells: bone marrow adiponectin-positive multinucleated cells take the lead
Source: Stem Cell Res Ther. 2023 Jul 4;14:173. doi: 10.1186/s13287-023-03400-w (PMC10320956; doi:10.1186/s13287-023-03400-w)
Supplement: Supplementary file 10 — Additional file 10. Fig. S5: Negative controls for the immunocytochemistry assay. LMCs stained with the isotype control antibodies were completely negative for the fluorescence signals. This observation validates the immunocytochemistry data demonstrated in Fig. 3. [file 13287_2023_3400_MOESM10_ESM.pdf]

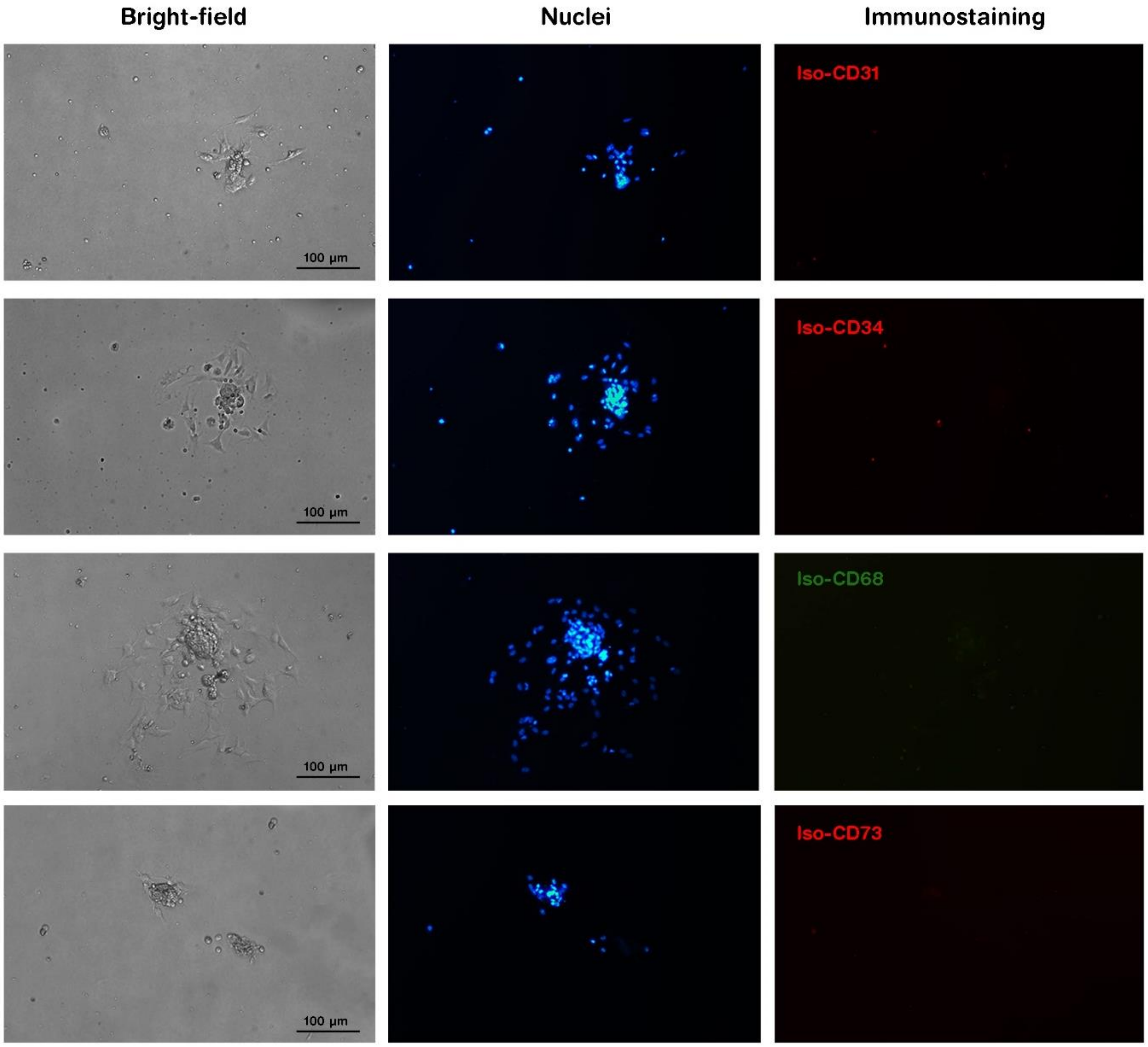

Supplementary figure 5: Negative controls for the immunocytochemistry assay. LMCs stained with the isotype control antibodies were completely negative for the fluorescence signals. This observation validates the immunocytochemistry data demonstrated in Fig. 3.
