# Supplementary material for: Regenerative potential of multinucleated cells: bone marrow adiponectin-positive multinucleated cells take the lead
Source: Stem Cell Res Ther. 2023 Jul 4;14:173. doi: 10.1186/s13287-023-03400-w (PMC10320956; doi:10.1186/s13287-023-03400-w)
Supplement: Supplementary file 11 — Additional file 11. Fig. S6: The expression of genes related to multinucleation. The bone marrow extracts were passed through a 100-µm pore size filter and then through a 20-µm filter. The cells that attached to the surface of the 20-µm mesh and those that passed through it were separately cultured for 24 h. Non-adherent cells and debris were washed, and the attached cells were used for RNA isolation and cDNA synthesis. Quantitative gene expression analysis on genes related to multinucleation demonstrated that the LMC-rich 20–100 µm fraction has substantially higher expression of Sox2, Cdk1, Chk1, and E2f1 compared to the other population that is rich of mononucleated cells. On the other hand, the genes such as Plk1, Nanog, Oct4, Sycp3, and Rec8 are down-regulated in multinucleated cells. [file 13287_2023_3400_MOESM11_ESM.pdf]

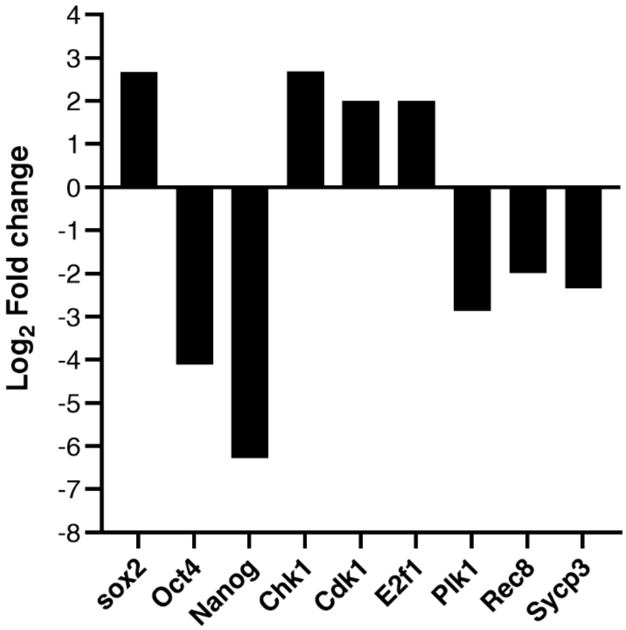

Supplementary Figure 6. The expression of *genes related to multinucleation*. The bone marrow extracts were passed through a 100 µm pore size filter and then through a 20 µm filter. The cells that attached to the surface of the 20 µm mesh (size: 20-100 µm) and those that passed through it (size < 20 µm) were separately cultured for 24 hours. Non-adherent cells and debris were washed and the attached cells were used for RNA isolation and cDNA synthesis. Quantitative gene expression analysis on genes related to multinucleation demonstrated that the LMC-rich 20-100 µm fraction has substantially higher expression of *Sox2, Cdk1, Chk1, and E2f1* compared to the other population that is rich of mono-nucleated cells. On the other hand, the genes *Plk1, Nanog, Oct4, Sycp3*, and *Rec8* are down-regulated in multinucleated cells.
