# Supplementary material for: Regenerative potential of multinucleated cells: bone marrow adiponectin-positive multinucleated cells take the lead
Source: Stem Cell Res Ther. 2023 Jul 4;14:173. doi: 10.1186/s13287-023-03400-w (PMC10320956; doi:10.1186/s13287-023-03400-w)
Supplement: Supplementary file 12 — Additional file 12. Fig. S7: Islands of LMCs during BM regeneration. In days 12 and 20 post-sub-lethal irradiation, LMC-rich foci are observed in BM sections. [file 13287_2023_3400_MOESM12_ESM.pdf]

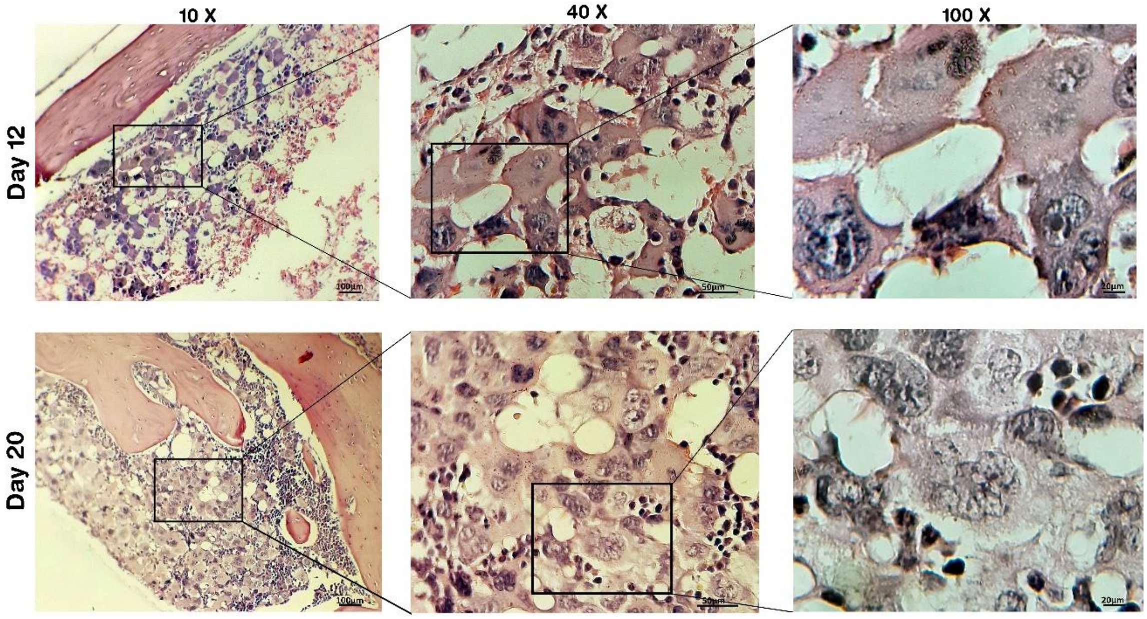

Supplementary Figure 7: Islands of LMCs during BM regeneration. In days 12 and 20 post sub-lethal irradiation, LMC-rich foci are observed in BM sections.
